# Vasculitis and Neutrophil Extracellular Traps in Lungs of Golden Syrian Hamsters With SARS-CoV-2

**DOI:** 10.3389/fimmu.2021.640842

**Published:** 2021-04-12

**Authors:** Kathrin Becker, Georg Beythien, Nicole de Buhr, Stephanie Stanelle-Bertram, Berfin Tuku, Nancy Mounogou Kouassi, Sebastian Beck, Martin Zickler, Lisa Allnoch, Gülsah Gabriel, Maren von Köckritz-Blickwede, Wolfgang Baumgärtner

**Affiliations:** ^1^ Department of Pathology, University of Veterinary Medicine Hannover, Hannover, Germany; ^2^ Department of Biochemistry, University of Veterinary Medicine Hannover, Hannover, Germany; ^3^ Research Center for Emerging Infections and Zoonoses (RIZ), University of Veterinary Medicine Hannover, Hannover, Germany; ^4^ Department for Viral Zoonoses-One Health, Heinrich Pette Institute, Leibniz Institute for Experimental Virology, Hamburg, Germany; ^5^ Institute for Virology, University for Veterinary Medicine Hannover, Hannover, Germany

**Keywords:** vasculitis, neutrophils extracellular traps (NETs), SARS-CoV-2, hamster, COVID-19

## Abstract

Neutrophil extracellular traps (NETs) have been identified as one pathogenetic trigger in severe COVID-19 cases and therefore well-described animal models to understand the influence of NETs in COVID-19 pathogenesis are needed. SARS-CoV-2 infection causes infection and interstitial pneumonia of varying severity in humans and COVID-19 models. Pulmonary as well as peripheral vascular lesions represent a severe, sometimes fatal, disease complication of unknown pathogenesis in COVID-19 patients. Furthermore, neutrophil extracellular traps (NETs), which are known to contribute to vessel inflammation or endothelial damage, have also been shown as potential driver of COVID-19 in humans. Though most studies in animal models describe the pulmonary lesions characterized by interstitial inflammation, type II pneumocyte hyperplasia, edema, fibrin formation and infiltration of macrophages and neutrophils, detailed pathological description of vascular lesions or NETs in COVID-19 animal models are lacking so far. Here we report different types of pulmonary vascular lesions in the golden Syrian hamster model of COVID-19. Vascular lesions included endothelialitis and vasculitis at 3 and 6 days post infection (dpi), and were almost nearly resolved at 14 dpi. Importantly, virus antigen was present in pulmonary lesions, but lacking in vascular alterations. In good correlation to these data, NETs were detected in the lungs of infected animals at 3 and 6 dpi. Hence, the Syrian hamster seems to represent a useful model to further investigate the role of vascular lesions and NETs in COVID-19 pathogenesis.

## Introduction

Coronavirus disease 2019 (COVID-19), caused by severe acute respiratory syndrome coronavirus-2 (SARS-CoV-2), a betacoronavirus and the etiological agent of a worldwide occurring pandemic, represents a global challenge for the health care system. Infection can result in asymptomatic, mild, severe or fatal disease characterized by interstitial pneumonia and/or acute respiratory distress syndrome (ARDS) (1–3). Reported vascular findings include vascular leakage, endothelialitis, thrombosis and angiogenesis in the lung. Pathological examinations of patients who died of COVID-19 indicate that vascular impairment and neutrophil extracellular trap (NET) formation seem to play a crucial role in fatal cases, in addition or in combination with various other host factors such as age, gender, immune status and comorbidities (1, 4–7). NETs are extracellular DNA fibers released by activated neutrophils during the innate immune response in animals and humans (8–10). They are described as an innate immune defense mechanism against bacteria, viruses, fungi and parasites (10–14). However, if NETs are not balanced in a host, they can become detrimental (15). They are described to be involved in thrombosis (16, 17), autoimmune diseases (18), stroke (19, 20) and other diseases (21, 22). A contribution of NETs to immunothrombosis in ARDS and in vascular occlusions was recently confirmed in COVID-19 patients (23, 24). Interestingly, NETs are described to be involved in the pathogenesis of ANCA-induced vasculitis (21, 25, 26) and may directly contribute to vessel inflammation by activating the complement system and inducing endothelial damage. Furthermore, treatment strategies with dornase alfa to destroy NETs in COVID-19 patients with ARDS are already discussed (27, 28). In addition, a study indicated that high titers of pro-thrombotic autoantibodies are associated with NETs in COVID-19 patients (29). High levels of NET markers were detected in serum samples of COVID-19 patients (30, 31) and, thus, it is very strongly hypothesized that NETs are involved in the pathogenesis of COVID-19 (28, 32, 33).

Many studies investigating COVID-19 pathogenesis referred to pulmonary lesions consisting of interstitial pneumonia, analyzed virus spread and investigated the impact of vaccination strategies on disease development in various animal models. However, vascular alterations are not studied in detail despite their pivotal role in fatal cases in humans, and vascular alterations are mentioned only infrequently in the animal models of COVID-19 (34–38). Furthermore, to unravel the role of NETs and the molecular mechanisms leading to NET-depending COVID-19 symptoms, animal models are still missing to study the pathogenesis and investigate new treatment strategies.

To develop new treatment strategies for SARS-CoV-2 infections and comparable diseases in the future, an understanding of the pathogenesis is needed. Next to analyzing human cohorts, animal models will help to understand complex host-pathogen interactions and provide a platform to investigate innovative treatment strategies in more detail. The comparison of observations in diseased humans and animal models will help to obtain better insights in the COVID-19 disease and pathogenesis. Interestingly, an analysis of SARS-CoV-2 infected humans and Rhesus Macaques showed an upregulation of pro-inflammatory cytokines, complement and coagulation cascade as well as endothelial damage and thrombosis. The authors underlined that the IL-6-JAK-STAT-3 pathway seems to play a crucial function on the immune system reaction during COVID-19 (39). The IL-6-JAK-STAT-3 pathway is known as a key regulator for the switch of neutrophils and therefore on NET release (40). However, so far no animal model is known that shows NET-formation and associated vasculitis to be involved in pathogenesis similar to the described NET-formation in lungs of severe cases of human SARS-CoV-2 infection. The golden Syrian hamster is described as one potential animal model for SARS-CoV-2 infections (41, 42). Furthermore, a study with *Entamoeba histolytica* identified NET formation in hamsters (9). Therefore, we hypothesized that the hamster is an animal model to investigate the immune-pathology of SARS-CoV-2 infections. Indeed, the present report shows for the first-time vasculitis and NETs in lungs of SARS-CoV-2 infected hamsters.

## Material and Methods

### Animals

All animal experiments were performed in strict accordance with the guidelines of German animal protection law and were approved by the relevant German authority (The local authority approval number: protocols N 32/2020). Male and female Syrian golden hamsters (8-10 weeks old) were purchased from Janvier Labs (Le Genest-Saint-Isle, France) and were kept under standard housing conditions (21 ± 2 °C, 40–50% humidity, food and water ad libitum) with a 12:12 light–dark cycle at the Heinrich Pette Institute, Leibniz Institute for Experimental Virology in Hamburg, Germany. Animals were intranasally infected with 10^5^ plaque forming units (p.f.u.) SARS-CoV-2 or mock infected with PBS under 150 mg/kg ketamine and 10 mg/kg xylazine anesthesia. Body weight was monitored daily over 14 days post infection (p.i.). At day 1, 3, 6 and 14 p.i. five female and five male animals per group were euthanized by intraperitoneal injection of an overdoses of pentobarbital and blood was drawn by cardiac puncture. The collected lungs were fixed in 4% paraformaldehyde for light microscopic examination.

### Virus

SARS-CoV-2 (SARS-CoV-2/Germany/Hamburg/01/2020; ENA study PRJEB41216 and sample ERS5312751) was isolated from a human nasopharyngeal swab of a confirmed patient with COVID-19 and propagated in Vero E6 cells in Dulbecco’s Modified Eagle’s Medium (DMEM) (Sigma-Aldrich GmbH) supplemented with 2% fetal bovine serum, 1% penicillin-streptomycin and 1% L-glutamine at 37°C. All infection experiments with SARS-CoV-2 were performed in the biosafety level 3 (BSL-3) laboratory at the Heinrich Pette Institute, Leibniz Institute for Experimental Virology in Hamburg, Germany.

### Histological Evaluation

The lung was immersion-fixed in 10% neutral-buffered formalin and routinely embedded in paraffin. 3 µm sections were stained with hematoxylin and eosin (HE) for examination by light microscopy.

The presence of vascular lesions was evaluated, included monitoring of vasculopathy with special emphasis on endothelialitis, vasculitis, and vascular wall degeneration. Statistical analysis was performed using Kruskal-Wallis test followed by Mann–Whitney U or Bonferroni *post hoc* test.

### Immunohistochemistry

For the detection of the SARS-CoV-2 antigen, following a modified and optimized version of the protocol published by Liu et al. (43), as well as the detection of the stable c fragment of complement component 3 (C3c) the EnVision+ detection system (Dako Agilent Pathology Solutions) was used. For the detection of clotting factor X (factor X) the avidin-biotin-peroxidase method was used. Sections of lung tissue were dewaxed and rehydrated in isopropanol and 96% ethanol followed by blockage of endogenous peroxidase by incubation in 85% ethanol with 0.5% H_2_O_2_ for 30 min at room temperature (RT). Antigen retrieval for the SARS-CoV-2 antigen was performed through incubation in citrate-Na_2_H_2_EDTA buffer (10 mM citric acid, 2 mM Na_2_H_2_EDTA, 0.05% Tween 20) for 20 minutes in a microwave at 800 W, followed by a 20 minute cooldown period at RT. For the staining of factor X sections were treated with proteinase K (Merk, 124568, 1:2000 in PBS) for 5 min at RT, while no antigen retrieval was performed for C3c detection. Sections were thereafter transferred to Shandon Coverplates™ (Thermo Electron GmbH) and sections for factor X detection were blocked for 30 minutes at RT with inactivated normal goat serum diluted 1:5 in PBS. For the detection of SARS-CoV-2 antigen, a monoclonal antibody against the SARS-CoV-2 NP (Sino Biological, 40143-MM05, 1:32,000) diluted in PBS containing 1% BSA with addition of 0,3% Triton 100 was applied for 1 h at RT. For the detection of C3c and factor X, a rabbit polyclonal antibody against human C3c (Dako Agilent Pathology Solutions, A0062, 1:200) or a mouse IgG2b antibody against factor X (Sigma Aldrich, F8396, 1:50) diluted in PBS containing 1% BSA were applied respectively overnight at 4°C. Sections were subsequently rinsed, and secondary labeling was performed using either the respective peroxidase-labeled polymer (Dako Agilent Pathology Solutions, K4001 and K4003; SARS-CoV-2 and C3c) for 30 minutes or a biotinolated goat anti-mouse antibody (Vector Laboratories Inc., BA-9200, 1:200; Factor X) for 60 minutes at RT. For factor X detection, a tertiary treatment with the avidin-biotin-peroxidase complex (Vectastain Elite ABC Kit, PK-6100, Vector Laboratories Inc) was performed according to the manufacturer’s instructions. Visualization of the reaction was accomplished by incubation in chromogen 3,3diaminobenzidine tetrahydrochloride (DAB, 0.05%) and 0.03% H2O2 in PBS for 5 min. The slides where afterwards counterstained with Mayer’s hematoxylin for 1 min. As negative control, the primary antibody was replaced with ascites fluid from Balb/c mice (1:1000, SARS-CoV-2 and Factor X) or rabbit normal serum (1:3000, C3c).

### NET Examination

For NET detection paraffin sections of lungs from all animals (3 dpi and 6 dpi) were analyzed. The immunofluorescence staining of paraffin section was performed as previously described (44) with the following changes. First mouse IgG2a anti-DNA/histone (Millipore MAB3864, 0.55 mg; 1:100) and rabbit anti-human myeloperoxidase antibodies (Dako A0398, 3.2 mg, 1:300) in blocking buffer were incubated overnight at 4°C. As secondary antibodies, a goat anti-mouse antibody (Alex488PLUS, Thermo Fisher Scientific) and goat-anti rabbit antibody (Alexa633, Thermo Fisher Scientific or Alexa568, Thermo Fisher Scientific) were used diluted 1:500 in blocking buffer.

The H3cit/DNA/histone1 staining was conducted with first antibodies mouse IgG2a anti-DNA/histone1 (Millipore MAB3864, 0.55 mg; 1:100) and rabbit anti-human H3cit (ABCAM ab5103, 1:25) in a blocking buffer as described previously (24) overnight at 4°C. As secondary antibodies, a goat anti-mouse antibody (Alex488PLUS, Thermo Fisher Scientific) and goat-anti rabbit antibody (Alexa568, Thermo Fisher Scientific) were used diluted 1:500 in blocking buffer.

The C3c/DNA/histone1 staining was conducted with first antibodies mouse IgG2a anti-DNA/histone1 (Millipore MAB3864, 0.55 mg; 1:100) and rabbit anti-human C3c complement (ABCAM A0062, 1:100) in blocking buffer described above for 1h at room temperature. As secondary antibodies, a goat anti-mouse antibody (Alex488PLUS, Thermo Fisher Scientific) and goat-anti rabbit antibody (Alexa568, Thermo Fisher Scientific) were used diluted 1:500 in blocking buffer.

The Factor X/myeloperoxidase staining was conducted with first antibodies mouse IgG2b anti-factor X (Sigma Aldrich, 1.0 mg/mL; 1:50) and rabbit anti-human myeloperoxidase antibodies (Dako A0398, 3.2 mg, 1:300) in blocking buffer described overnight at 4°C. As secondary antibodies, a goat anti-mouse antibody (Alex488PLUS, Thermo Fisher Scientific) and goat-anti rabbit antibody (Alexa568, Thermo Fisher Scientific) were used diluted 1:500 in blocking buffer.

At the end, all samples were processed with TrueVIEW autofluorescence quenching kit (Vector laboratories) following the manufacturer’s instructions.

Samples were recorded using a Leica TCS SP5 AOBS confocal inverted-base fluorescence microscope with HCX PL APO 40 × 0.75–1.25 oil immersion objective or HCX PL APO 63 × 1.40 oil (C3c/DNA/histone). The settings were adjusted using isotype control antibodies in separate preparations.

In all 10 animals 3 dpi (mock and SARS-CoV-2 infected) and all 10 animals 6 dpi (SARS-CoV-2 infected) one section was stained and for 5 minutes screened for NET-positive areas. At least 3 pictures were generated from each sample and analyzed for the number of NET-positive areas. The mean of NET-positive areas was calculated for the analyzed area.

For statistical analysis with an unpaired one-tailed Mann-Whitney test was applied.

Sections were analyzed as serial cuts (4 µm each) for NETs (overlay), Hematoxylin-Eosin (H&E) staining, and virus protein.

### DNase *In Vitro* Digestion of NETs

During the staining of paraffin sections, a DNase digestion step was included directly before the first antibody was added. A DNase activity buffer (pH 7.4, 3mM CaCl_2_, 3mM MgCl_2_, 300mM Tris) was mixed with a DNase cocktail (micrococcal nuclease, 100 mU final, Sigma Aldrich and Deoxyribonuclease I from bovine pancreas, 20 U final, Sigma Aldrich) and the sections were incubated with this mix in a humid chamber at 37°C for 30 minutes. Afterwards sections were washed, and the staining protocol continued as described above. A respective isotype control was undergoing the same procedure. The DNase treated sections were compared with the respective serial cuts.

## Results and Discussion

### Endothelialitis and Vasculitis in Lungs of Golden Syrian Hamster (Hamster) At 3 and 6 Days Post Infection (dpi)

**Figure 1:**
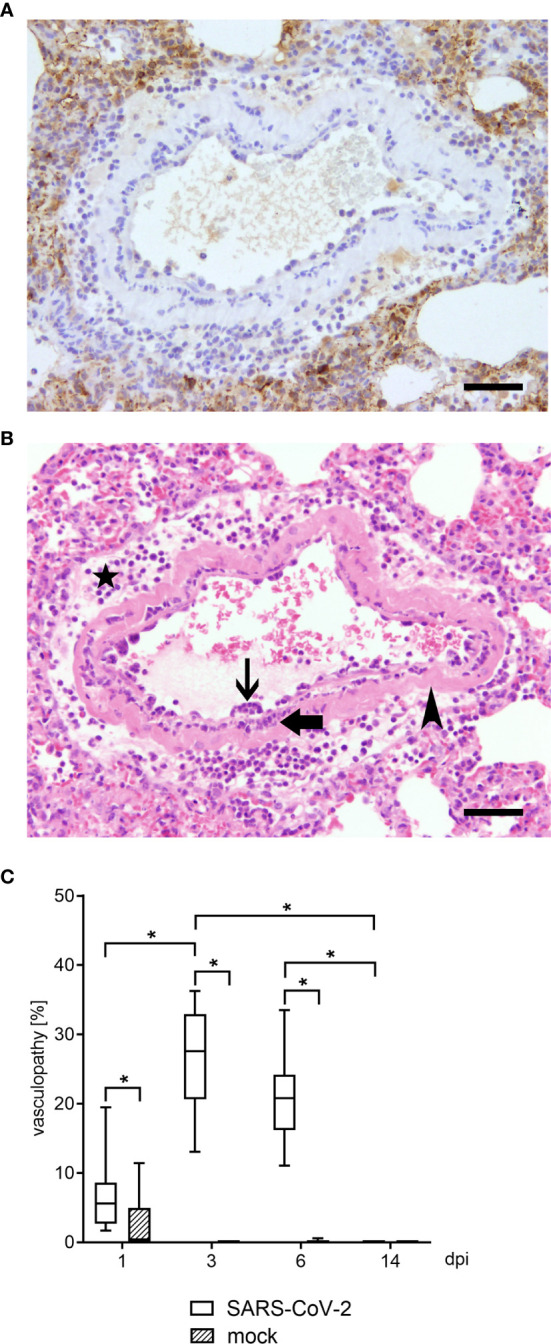
Vascular lesions and virus protein were identified by histological and immunohistological examination in lungs of hamsters 3 dpi. Representative pictures of light microscopic pulmonary findings are presented (3 dpi). **(A)** While viral antigen expression was detected in lung lesions (dark brown coloration), none was present in affected blood vessels. Immunohistochemistry for SARS-CoV-2 nucleoprotein, avidin–biotin complex -method, 3,3’-diaminobenzidine, light microscopy, scale bar 50 µm. **(B)** An affected blood vessel shows endothelium which is bulging into the lumen due to an infiltration by macrophages and lymphocytes (endothelialitis; thin arrow). Further, inflammatory cells, mainly macrophages and neutrophils were present within the vessel wall (vasculitis; thick arrow). Segmentally, the vessel wall shows hypereosinophilia and loss of cellular details (arrowhead). Additionally, perivascular edema and perivascular infiltrates, consisting of macrophages and neutrophils, were present (star). Hematoxylin and eosin stain. 200 x. Hematoxylin and eosin, light microscopy, scale bar 50 µm. **(C)** Vasculopathy including endothelial hypertrophy, endothelialitis and vasculitis was quantified within the lungs of SARS CoV-2 or mock infected animals using hematoxylin and eosin (H&E) staining. At 1, 3 and 6 dpi SARS CoV-2 infected animals showed a significant increase of vascular alterations compared to controls. Furthermore, vasculopathy was significantly increased at 3 dpi compared to 1 and 14 dpi in SARS-CoV-2 infected hamsters. Also, at 6 dpi vascular alterations remained increased compared to 14 dpi. At 14 dpi no vasculopathy could be observed. Data are shown as box-and-whisker plots with mean and quartiles. Significant differences between the groups obtained by Kruskal-Wallis test followed by Mann–Whitney U or Bonferroni *post hoc* test were indicated by *p < 0.05.

The golden Syrian hamster (hamster) is described as one potential very useful animal for SARS-CoV-2 infections (35). Subsequently, we infected intranasally hamsters with SARS-CoV-2 to investigate pulmonary lesions. The infection was confirmed by intralesional detection of viral antigen *via* immunohistochemistry (**Figure 1A**).

Histologically, varying degrees of inflammatory changes, mainly infiltration of macrophages and neutrophils, affecting airways, alveolar spaces and interstitium were observed at all time points. In addition, four different types of vascular lesions occurred (separately or combined): (A) A prominent feature in multiple blood vessels was the presence of inflammatory cells, mainly macrophages and lymphocytes, beneath or within the endothelial cell layer (endothelialitis, **Figure 1B**). Single inflammatory cells could also be found close to the endothelium within the lumen. (B) Furthermore, endothelial cells were frequently bulging into the lumen (endothelial hypertrophy). (C) Vessel walls were also infiltrated by inflammatory cells, namely neutrophils, macrophages and lymphocytes (vasculitis, **Figure 1B**). (D) Rarely, blood vessel walls showed multifocal hypereosinophilia and loss of cellular detail (mural vascular wall degeneration, **Figure 1B**). The percentage of blood vessels affected by vascular lesions, summarized under the term vasculopathy, was statistically analyzed, and revealed a significant number of affected vessels from 1 to 6 dpi in infected animals compared to controls (**Figure 1C**). In association with the vascular lesions, moderate to severe perivascular edema as well as perivascular infiltrates, consisting mainly of macrophages, were present. Moreover, its severity increased from day 1 to day 6. At 14 dpi, single macrophage-like cells within vessel walls and minimal perivascular cellular infiltrates were the only residual vessel-associated change in infected animals. Viral antigen could be demonstrated *via* immunohistochemistry in various cell types, including epithelial cells and macrophage cells at 1, 3 and 6 dpi. At 14 dpi no viral antigen expression could be detected in any lung region. At no time point viral antigen was detected within lesioned or intact blood vessels (**Figure 1A**).

### Frequently Detected NETs in Lung Tissue of SARS-CoV-2 Infected Golden Syrian Hamsters 3 and 6 dpi

**Figure 2:**
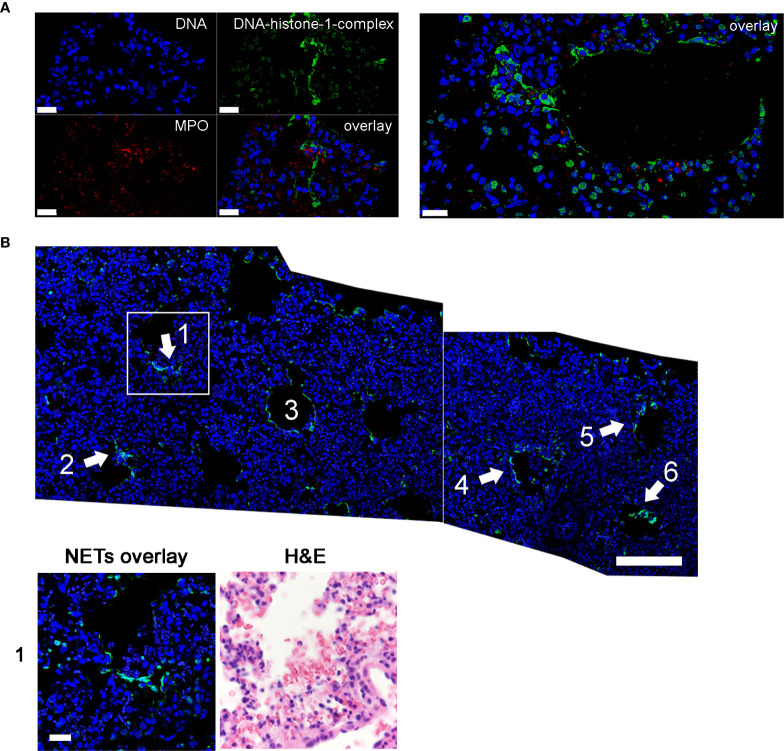
NETs as extracellular DNA-fibers were detected in lungs of SARS-CoV-2 infected hamsters 3 dpi by confocal immunofluorescence microscopy. **(A)** NETs as extracellular DNA-fibers were detected 3 dpi in lungs of SARS-CoV-2 infected hamsters. Representative 3D images of z-stacks were constructed with LAS X 3D Version 3.1.0 software (Leica) (upper panel: 3.36 µm consisting of 21 sections, lower panel: 4.25 µm consisting of 25 sections). **(B)** Stitched images show distribution of NETs (area 1–5) in the lung of SARS-CoV-2 infected hamster (blue = DNA, green = DNA-histone-1-complex). A zoom picture of area 1 is presented. Serial cuts were stained for NETs and H&E and allowed identification of blood vessels. NETs can be found interstitial and intrabronchial. Scale bars: stitched image = 100µm, magnification image = 20 µm. Settings of the immunofluorescence microscope were adjusted to a respective isotype control.

**Figure 3:**
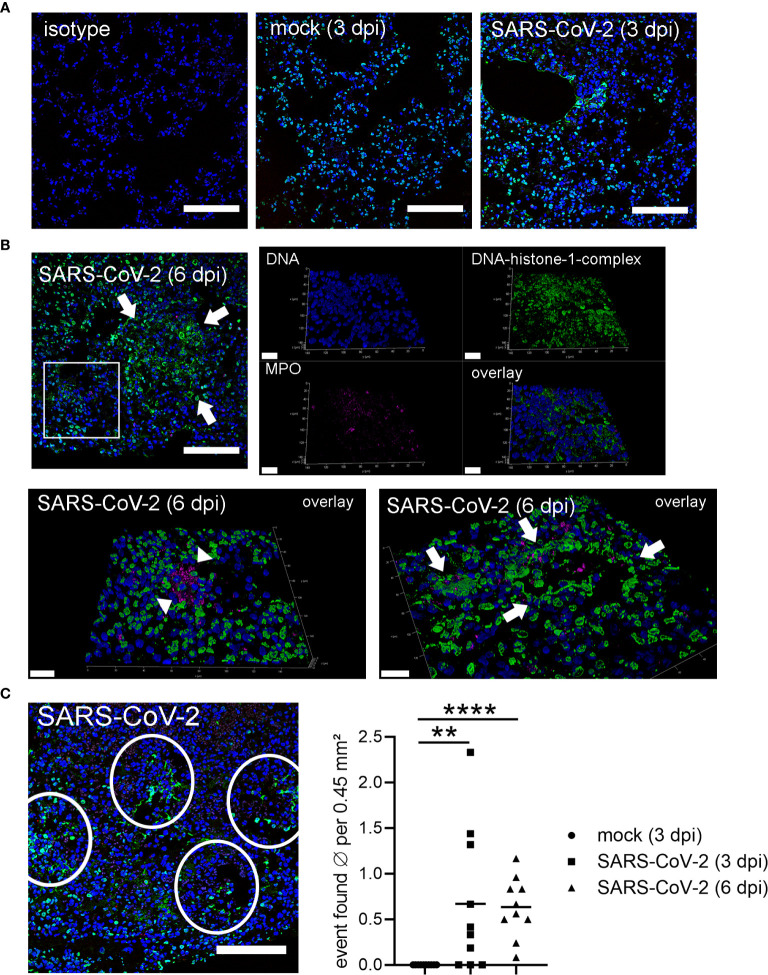
Detection of NETs in lungs only in SARS-CoV-2 infected hamsters. **(A)** Lung sections (3 dpi) form the non-infected group (mock) and infected group (SARS-CoV-2) were analyzed by confocal immunofluorescence microscopy for NET structures. The settings were adjusted to a respective isotype control. Representative images are presented (blue = DNA, green = DNA-histone-1-complex). **(B)** Lung sections (6 dpi) from the infected group (SARS-CoV-2) were analyzed for NET structures and NETs were detected. Representative 3D images of z-stacks were constructed with LAS X 3D Version 3.1.0 software (Leica) (upper panel: 9.57 µm consisting of 58 sections, lower panel left side: 12.25 µm consisting of 74 sections, lower panel right side: 8.22 µm consisting of 50 sections). Arrows show NET structures; arrow heads show myeloperoxidase (MPO). Representative images are presented (blue = DNA, green = DNA-histone-1-complex, magenta = MPO). Scale bars in all 3D images = 20 µm, scale bar 2D image = 100µm. **(C)** Semiquantitative analysis of NET formation in SARS-CoV-2-infected animals (3 dpi and 6 dpi) was significantly increased compared to mock animals. One representative image for counting events is shown. The mean of NET-positive areas was calculated for the analyzed area. The statistical analysis was calculated with an unpaired one-tailed Mann-Whitney test (**p = 0.0015; ****p < 0.0001). Scale bar = 100 µm. Settings of the immunofluorescence microscope were adjusted to a respective isotype control.

Interestingly, at 3 dpi we could demonstrate infiltrating neutrophils into the lung of SARS-CoV-2 infected hamsters and the release of NETs. DNA-fibers consisting of DNA-histone complexes and associated myeloperoxidase, which is a typical characteristic for NETs, were found interstitially (**Figure 2A**) (10, 45). NET-formation could be found frequently in lung areas with inflammatory infiltrates, consisting of macrophages and neutrophils, within alveoli and interstitium (**Figure 2**). NET markers cannot be found in the lungs of uninfected hamsters (**Figure 3A**), however NET markers were found in SARS-CoV-2 infected hamsters 6 dpi with a tendency to cover a larger area instead of more single events per view field (**Figure 3B**). A semiquantitative analysis of NET formation revealed significantly higher NET-formation in SARS-CoV-2-infected animals 3 and 6 dpi compared to uninfected animals. The observation showed a more even picture in the hamsters 6 dpi (**Figure 3C**).

**Figure 4:**
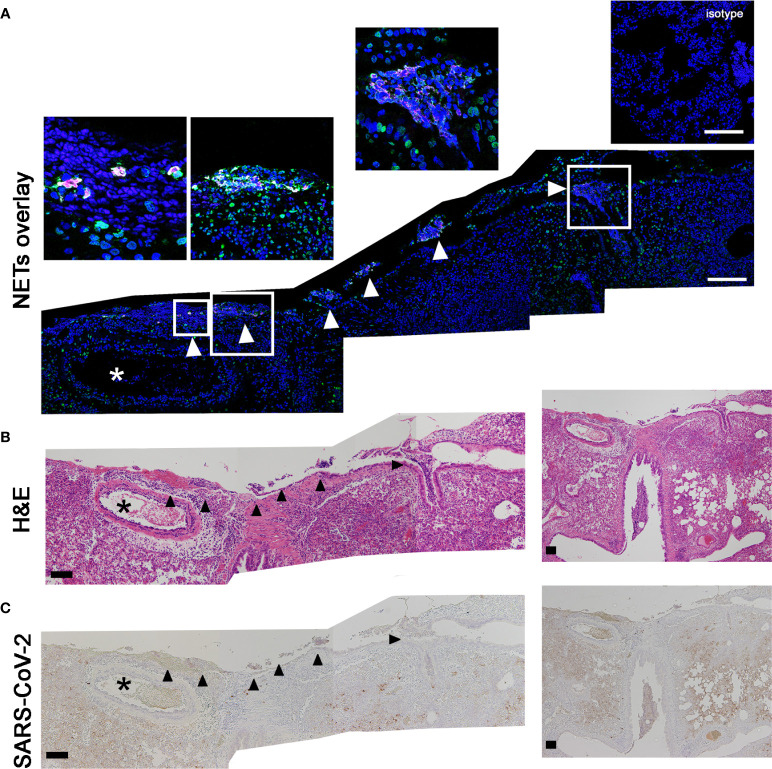
H3cit positive NET structures are found close to vessels with vasculopathy and SARS-CoV-2 protein. **(A)** The staining of citrullinated histones (H3cit = magenta) and DNA-histon-1-complexes (green) as NET-marker was conducted. The DNA is stained in blue. **(B)** The two following serial cuts were stained for H&E and **(C)** virus antigen (dark brown coloration). Events of NETs are detected inside of broncholi (arrow heads). Blood vessel (asterisk) with vasculitis were found close to NET positive areas. Scale bars: all stitched images **(A–C)** = 100 µm. Settings of the immunofluorescence microscope were adjusted to a respective isotype control.

Thus, we were interested if the NET formation is associated to the identified vascular lesions and may represent the triggering pathogenetic mechanism. Therefore, we analyzed serial cuts of infected hamsters with frequently detected NET formation inside the lung for NET formation (**Figure 4A**), associated pathological changes (**Figure 4B**) and virus distribution (**Figure 4C**). Interestingly, the NET structures were positive for citrullinated histones besides DNA-histone complexes. Citrullinated histones (H3cit) are described as key marker for NET formation (46). The analysis of the serial cuts allowed us to correlate the regions with NET positive structures in the lung tissue with histiocytic and neutrophilic infiltrates. NET structures were identified inside the bronchioli of SARS-CoV-2 infected hamsters and in close localization to blood vessel and damaged lung tissue (**Figure 4B**). Finally, the SARS-CoV-2 protein was detected in a serial cut. Therefore, three different markers for NETs (histone, MPO and citrullinated histone) were identified in the lungs of infected hamsters.

It was recently shown that NETs may trigger vasculitis (25) and can lead to vascular barrier injury (22).

Skendros et al., recently showed that complement and tissue factor were identified as a key driver in COVID-19 severity: (47). Tissue factor (TF) is initiating downstream cascades involving factor X (48). Here, we confirm that SARS-CoV-2-infected hamster exhibit distinct factor X as well as complement C3c protein level in inflamed lung biopsies (**Supplementary Figures 1–4**). Unfortunately, we were not able to confirm TF expression, since antibody-based staining of hamster biopsies did not show a distinct signal compared to respective isotype control samples.

**Figure 5:**
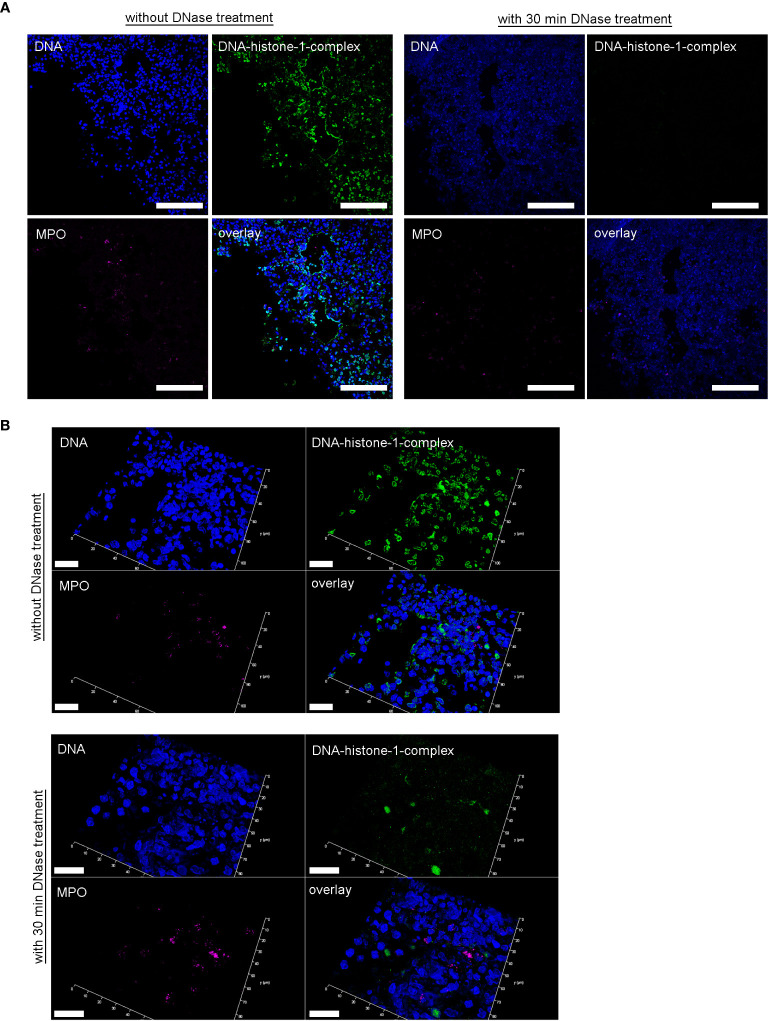
DNase treatment of 30 minutes delete the DNA-histone-1-complex from lung sections, but not the myeloperoxidase. **(A)** Serial cuts of lungs from SARS-CoV-2 infected hamsters were treated during the NET-staining with and without DNase for 30 minutes at 37°C. The settings were adjusted to a respective isotype control (with and without DNase treatment). **(B)** Representative 3D images of z-stacks from the same localization in two serial cuts (with and without DNase treatment) were constructed with LAS X 3D Version 3.1.0 software (Leica) (upper panel: 4.36 µm consisting of 27 sections, lower panel: 6.55 µm consisting of 40 sections). Representative images are presented (blue = DNA, green = DNA-histone-1-complex, magenta = MPO). Scale bars in **(A)** = 100 µm, in **(B)** = 20µm.

Furthermore, in severe COVID-19 patients an IgG fraction with high titers of autoantibodies targeting phospholipids and phospholipid-binding proteins were identified to trigger NET release (29). This underlines the complex involvement of NET-formation in the pathogenesis of severe COVID-19 infections. It is known that NET-associated histones, proteases and antimicrobial components lead to pore-formation and cytotoxicity based on their cationic charge and subsequent affinity to bind phospholipid-membranes (10, 49, 50). Therefore, an uncontrolled or overexpressed NET-formation can increase vascular permeability, lead to loss of barrier integrity, and may act cytotoxic. The detected microvascular injury during SARS-CoV-2-infections can at least partially be explained by these findings and is substantiated by recent observations of increased NET markers in the blood of SARS-CoV-2 patients (23). DNases can efficiently destroy NETs, and therefore clinical trials applying DNase treatment as potential therapeutic intervention in severe COVID-19 patients may be warranted (51). Furthermore, treatment strategies with dornase alfa to destroy NETs in COVID-19 patients with ARDS are already investigated in clinical trials (27, 51). Here we confirmed in a control experiment, that *ex vivo* nuclease treatment of lung biopsies from SARS-CoV-2-infected hamster successfully degraded present NETs (**Figure 5**).

In conclusion, the Syrian hamster seems to represent one possible animal model for SARS-CoV-2 infections that closely mimics the vascular lesions and NET-formation observed in humans. Though vascular lesions were detectable in the acute phase of the infection only, the type of lesion, vascular wall involvement, and cellular characteristics reflect what has been described in humans. Most importantly the lack of vascular lesion associated SARS-CoV-2 protein expression indicates a potentially indirect, presumably virus triggered, mechanism, e.g. cytokine release, which might also account for vascular lesions in humans. The present report shows for the first time in detail virus associated vascular lesions in an appropriate animal model, which will allow to further investigate the pathogenesis of SARS-CoV-2 associated vascular lesions as well as formation of NETs which might represent a key pathogenetic feature of severe cases in COVID-19 patients.

In future studies, it is needed to detect and quantify NET products and possible associated NET-specific autoantibodies as late-term consequence of NET associated pathology in this and other animal models to see if they might also allow to study detrimental effects of autoantibodies as long-term sequelae. Furthermore, long-term experiments are needed to investigate if the hamster is for example an useful animal model to investigate the influence of autoantibodies targeting phospholipids and phospholipid-binding proteins on NET-formation and thrombo-inflammatory processes during SARS-CoV-2 infection (29).

Our data suggest that NETs already during the early infection phase with SARS-CoV-2 are involved in the vascular pathology. Therefore, it is conceivable that even later occurring NET associated pathologies in the blood vessels may be investigated in the hamster model. However, this requires future research using the hamster as a model.

In summary, NETs and vasculopathy are part of the pathogenesis in severe COVID-19 patients. Animal models to understand the pathogenesis are urgently needed. The present observation shows for the first-time formation of NETs in lungs of SARS-CoV-2 infected golden Syrian hamster close to vessels with vasculopathy and SARS-CoV-2 protein three and 6 days post infection (dpi). The appearance of the vasculopathy was recorded over a period of 14 days but decreased after 6 dpi. Therefore, it seems that the golden Syrian hamster represents an animal model to understand the pathogenesis associated with NETs and vasculitis during SARS-CoV-2 infection, and to test new treatment strategies to counteract the severe effects during SARS-CoV-2 infection in lungs. The SARS-CoV-2-infection hamster model allows to study the mechanistical relation of NETs, vasculitis and COVID-19 pathogenesis at the starting point of infection and therefore to test early intervention treatment strategies of COVID-19.

## Data Availability Statement

The original contributions presented in the study are included in the article/**Supplementary Material**. Further inquiries can be directed to the corresponding authors.

## Ethics Statement

All animal experiments were performed in strict accordance with the guidelines of German animal protection law and were approved by the relevant German authority (The local authorities’ approval number: protocols N 32/2020).

## Author Contributions

SS-B, BT, NK, MZ and GG planned and conducted the animal experiment. KB, SS-B, BT and NK performed necropsies. KB, GB, LA, and WB conducted histological and immunohistological analysis. NB and MK-B performed NET experiments and conducted NETs analysis. KB, GB, NB, MK-B, and WB analyzed and interpreted the results. KB, GB, NB, MK-B, and WB drafted the manuscript. All authors revised the manuscript. All authors contributed to the article and approved the submitted version.

## Funding

This study was in part supported by a BMBF (Federal Ministry of Education and Research) project entitled RAPID (Risk assessment in re-pandemic respiratory infectious diseases), 01KI1723G and by the Ministry of Science and Culture of Lower Saxony in Germany (14 - 76103-184 CORONA-15/20). This study was furthermore in part funded by the Covid-19 Rapid Response Grant of the German Ministry of Health (BMG) to GG. This publication was supported by the DFG and the University of Veterinary Medicine Hannover, Foundation within the funding program Open Access Publishing.

## Conflict of Interest

The authors declare that the research was conducted in the absence of any commercial or financial relationships that could be construed as a potential conflict of interest.
