# Supplementary material for: Vasculitis and Neutrophil Extracellular Traps in Lungs of Golden Syrian Hamsters With SARS-CoV-2
Source: Front Immunol. 2021 Apr 12;12:640842. doi: 10.3389/fimmu.2021.640842 (PMC8072219; doi:10.3389/fimmu.2021.640842)
Supplement: Supplementary file 1 [file DataSheet_1.pdf]

Supplementary Material

Vasculitis and neutrophil extracellular traps in lungs of golden Syrian hamsters with SARS-CoV-2

Kathrin Becker[1#], Georg Beythien[1#], Nicole de Buhr[2,3#], Stephanie Stanelle-Bertram[4], Berfin Tuku[4], Nancy Mounogou Kouassi[4], Sebastian Beck[4], Martin Zickler[4], Lisa Allnoch[1], Gülsah Gabriel[4,5], Maren von Köckritz-Blickwede[2,3*§], Wolfgang Baumgärtner[1*§]

[1]Department of Pathology, University of Veterinary Medicine Hannover, Hannover, Germany;

[2]Department of Biochemistry, University of Veterinary Medicine Hannover, Hannover, Germany;

[3]Research Center for Emerging Infections and Zoonoses (RIZ), University of Veterinary Medicine Hannover, Hannover, Germany;

[4]Heinrich Pette Institute, Leibniz Institute for Experimental Virology, Hamburg, Germany;

[5]Institute for Virology, University for Veterinary Medicine Hannover, Hannover, Germany;

#These authors contributed equally to this work as first authors.

§These authors contributed equally to this work as last authors.

*** Correspondence:**
Prof. Dr. Wolfgang Baumgärtner, Department of Pathology, University of Veterinary Medicine Hannover, Germany; Bünteweg 17, 30559 Hannover, Germany; phone: +49-511-953-8620; mail: Wolfgang.baumgaertner@tiho-hannover.de

Prof. Dr. Maren von Köckritz-Blickwede, Department of Biochemistry, University of Veterinary Medicine Hannover, Bünteweg 17, 30559 Hannover, Germany; phone: +49-511-953-8787; mail: mkoeckbl@tiho-hannover.de

Supplementary Figures

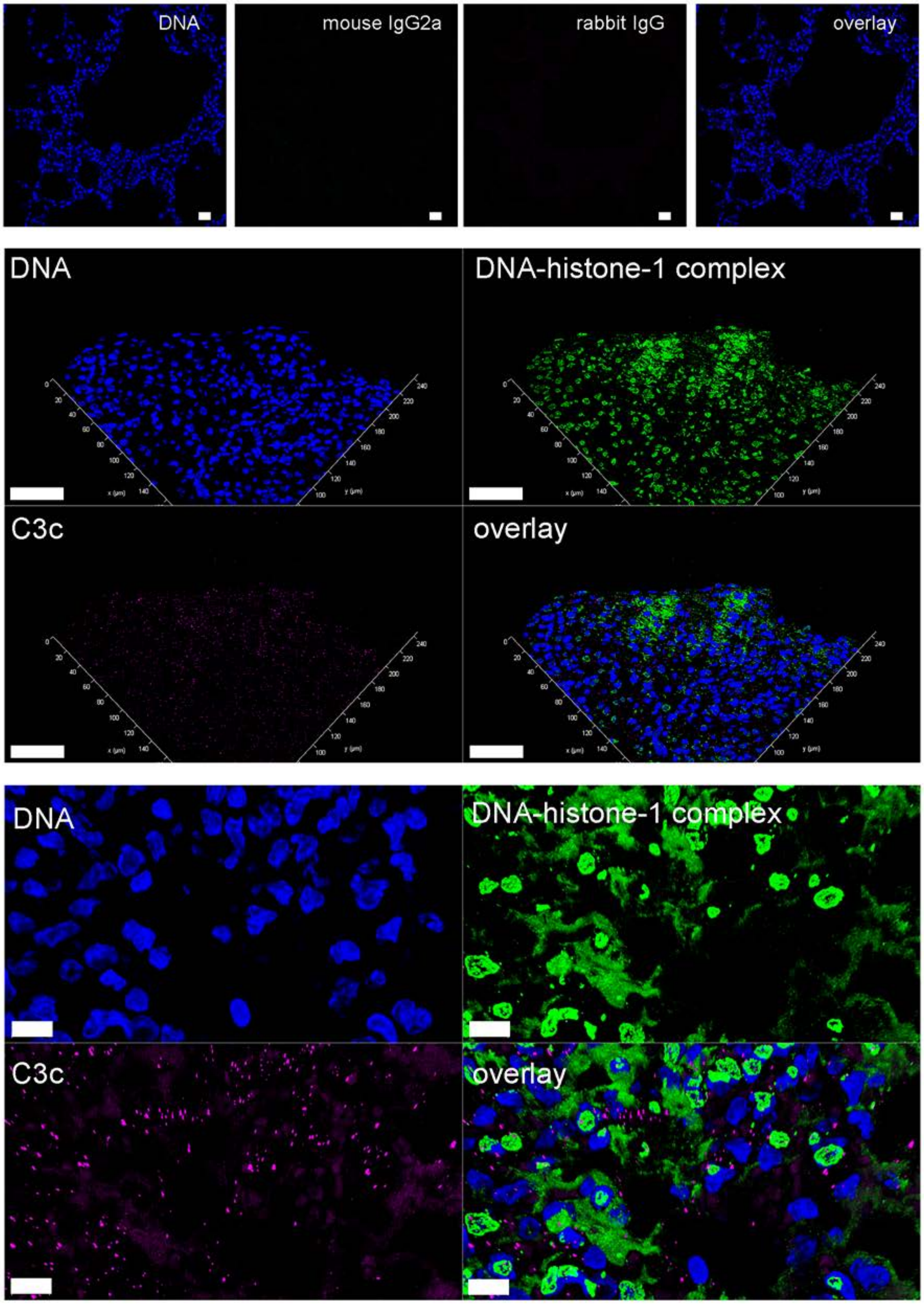

Supplementary Figure 1. C3c (complement) and positive NET structures are found in inflamed lung tissue of SARS-CoV-2 infected lungs.

(A) The staining of C3c (magenta) and DNA-histon-1-complexes (green) as NET-marker was conducted. The DNA is stained in blue. The upper panel shows the respective isotype control, and the settings of the immunofluorescence microscope were adjusted to this control. Representative 3D images of z-stacks from animals 6 dpi were constructed with LAS X 3D Version 3.1.0 software (Leica) (central panel: 2.77 µm consisting of 23 sections, lower panel: 6.38 µm consisting of 39 sections).

The presented representative images are serial cuts of the animal 3 and 4 in Supplemental Figure 2 and 4.

Representative images are presented. Scale bars in (A) upper panel = 20 µm, central panel = 50 µm, lower panel = 10µm. in (B) = 20µm.

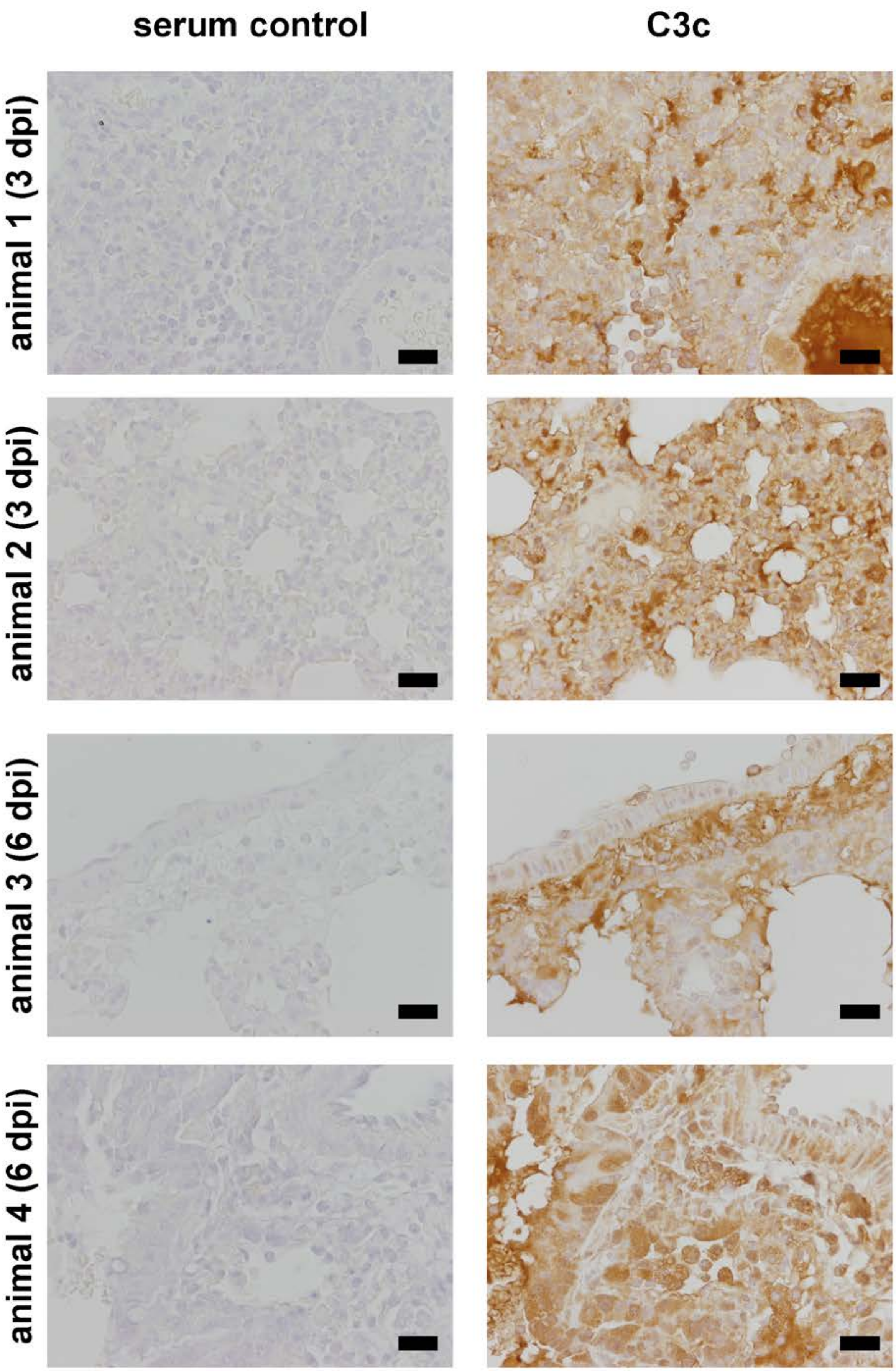
serum control
C3c
animal 1 (3 dpi)
animal 2 (3 dpi)
animal 3 (6 dpi)
animal 4 (6 dpi)

Supplementary Figure 2. C3c (complement) are found in inflamed lung tissue of SARS-CoV-2 infected lungs 3 and 6 dpi by immunohistological examination.

Immunohistochemical staining for C3c was conducted to evaluate the presence of the complement component 3 in SARS-CoV-2 infected animals. Images show representative lung lesions at 3 and 6 dpi. At both time points a strong signal was detected diffusely in bloodserum and edema fluid as well as disseminated on the surface of endothelial cells, bronchiolar epithelium, type 1 pneumocytes and macrophages. The left panel shows serum treated control sections to exclude nonspecific polymer binding. Bars represent 20µm.

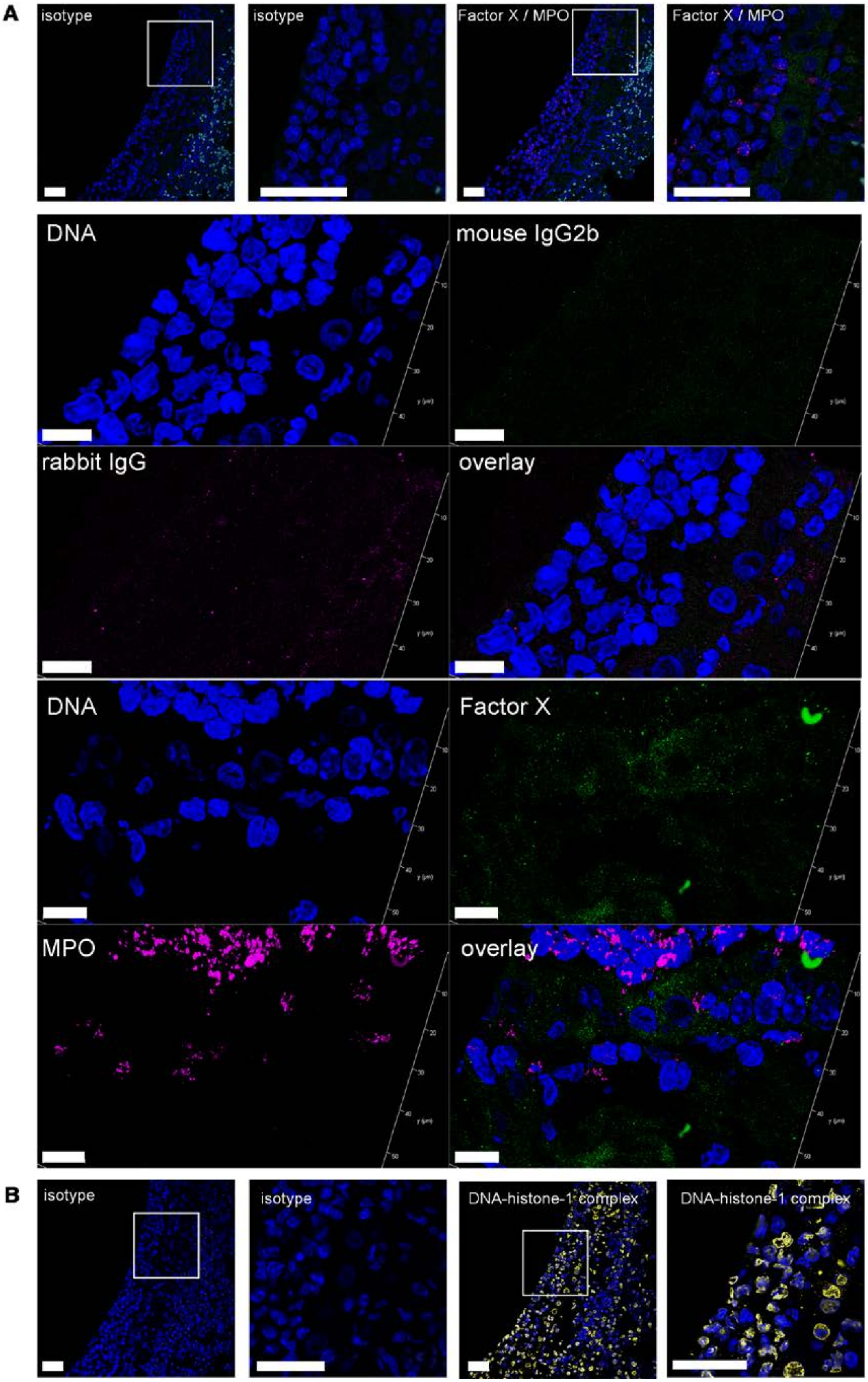
A
isotype
isotype
Factor X / MPO
Factor X / MPO
DNA
mouse IgG2b
rabbit IgG
overlay
DNA
Factor X
MPO
overlay
B
isotype
isotype
DNA-histone-1 complex
DNA-histone-1 complex

Supplementary Figure 3. Factor X and myeloperoxidase are found in lung tissue of SARS-CoV-2 infected lungs by immunofluorescence microscopy.

(A) The staining of Factor X (green) and myeloperoxidase (MPO = magenta) was conducted. The DNA is stained in blue. The upper panel shows on the left side the respective isotype control (overlay image of an overview and zoom). The upper panel shows on the right side the same area imaged in a serial cut for Factor X and myeloperoxidase (overlay image of an overview and zoom). The settings of the immunofluorescence microscope were adjusted to this control. Representative 3D images of z-stacks from a SARS-CoV-2 infected animal 3 dpi were constructed with LAS X 3D Version 3.1.0 software (Leica). The z-stacks were made at the same position as the 2D image shown in the upper panel (central panel: 4.28 µm consisting of 35 sections, lower panel: 4.78 µm consisting of 39 sections).

(B) The staining of DNA-histone-1-complex (yellow) was conducted in serial cuts of images presented in (A). The DNA is stained in blue. The left side shows the respective isotype control (overlay image of an overview and zoom). The right side shows the same area imaged DNA-histone-1-complex (overlay image of an overview and zoom).

The presented representative images are serial cuts of the animal 2 in Supplemental Figure 2 and 4.

Scale bars in (A) upper panel = 25 µm, central and lower panel = 10 µm, (B) = 25µm.

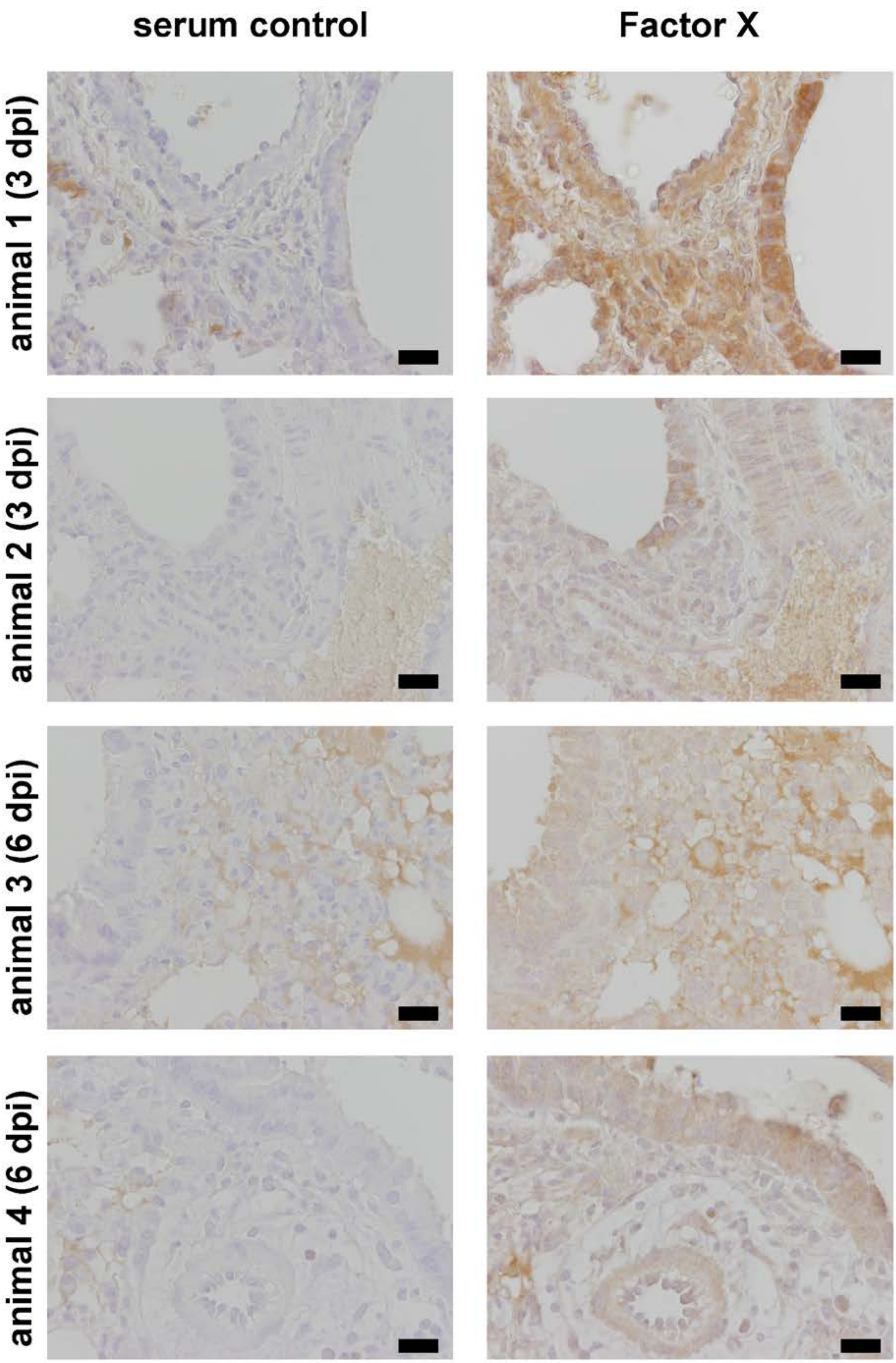
serum control
Factor X
animal 1 (3 dpi)
animal 2 (3 dpi)
animal 3 (6 dpi)
animal 4 (6 dpi)

Supplementary Figure 4. Factor X are found in lung tissue of SARS-CoV-2 infected lungs 3 and 6 dpi by immunohistological examination.

Immunohistochemistry was applied to visualize the distribution of factor X in SARS-CoV-2 infected hamsters. Images show representative lung lesions at 3 and 6 dpi. At both time points signal was obtained moderately in blood serum and edema fluid as well as mildly to moderately in the bronchial epithelium. Furthermore, animal 1 showed an overall stronger signal with additional staining of the vascular wall and the pulmonary interstitium, indicating individual variability in clotting factor expression. The left panel displays serum treated control sections to exclude nonspecific secondary antibody binding. Bars represent 20µm.
